# How does the clinical practice of Aotearoa New Zealand podiatrists align with international guidelines for the prevention of diabetes-related foot disease? A cross-sectional survey

**DOI:** 10.1186/s13047-023-00651-x

**Published:** 2023-08-22

**Authors:** Hannah Jepson, Peter A Lazzarini, Michele Garrett, Matthew R Carroll

**Affiliations:** 1 Department of Podiatry, School of Clinical Sciences, Faculty of Health & Environmental Sciences, Auckland University of Technology, Private Bag 92006, Auckland, 1142 New Zealand; 2 School of Public Health and Social Work, Queensland University of Technology, Brisbane, QLD Australia; 3 Metro North Hospital and Health Service, Allied Health Research Collaborative, Brisbane, QLD Australia; 4 Community and Long Term Conditions Directorate, Te Toka Tumai, Auckland, New Zealand

**Keywords:** Diabetes mellitus, Guidelines, Prevention, Foot

## Abstract

**Background:**

Given the importance of preventive care for the lower limb in people with diabetes, and the absence of local guidelines in Aotearoa New Zealand (NZ), the aim of this study was to determine the alignment of assessment and management used in the prevention of diabetes-related foot disease by NZ podiatrists to the international prevention guideline recommendations.

**Methods:**

A 37-item web-based survey was developed using a 5-point Likert scale (0 = always; 5 = never) based on the International Working Group of the Diabetic Foot (IWGDF) 2019 prevention guidelines and included domains on participant demographics, sector, caseloads, guidelines, screening, management, education, and referral. The survey was distributed to NZ podiatrists through the NZ podiatry association and social media. Participants completing > 50% of items were included. The Mann-Whitney U test was used to examine differences between sector subgroups.

**Results:**

Seventy-seven responses (16.3% of the NZ podiatry workforce) were received, of which 52 completed > 50% of items and were included. Of those 52 podiatrists, 73% were from the private sector. Public sector podiatrists reported higher weekly caseloads of patients with diabetes (*p* = 0.03) and foot ulcers (*p* < 0.001). The New Zealand Society for the Study of Diabetes (NZSSD) risk stratification pathway and IWGDF guidelines were the two most frequently utilised guidance documents. Participants reported median scores of at least “often” (< 2) for all items in the assessment and management, inspection, examination, and education provision domains for people with a high-risk foot. More than 50% of respondents reported screening more frequently than guideline recommendations for people with a very low to moderate risk foot. Structured education program was only used by 4 (5%) participants. Public sector podiatrists reported greater provision of custom-made footwear (*p* = 0.04) and multi-disciplinary team care (*p* = 0.03).

**Conclusion:**

NZ podiatrists generally follow international guideline recommendations with respect to screening, self-care education, appropriate footwear, and treatment of risk factors for people at-risk of diabetes-related foot disease. However there may be over-screening of people with very low to medium risk occurring in clinical practice. Increasing access to integrated healthcare, custom-made footwear and structured educational programmes appear to be areas of practice that could be developed in future to help prevent diabetes-related foot disease in NZ.

**Supplementary Information:**

The online version contains supplementary material available at 10.1186/s13047-023-00651-x.

## Background

 Diabetes is a leading and rapidly growing cause of the global disease burden and affects approximately 10.5% of the adult population worldwide [1]. In Aotearoa New Zealand (NZ), type 2 diabetes is on a trajectory to reach epidemic proportions within the next 20 years with the cost to the health system estimated to increase by 63% to $NZ 3.5 billion [2]. Diabetes-related foot disease (DFD) is a leading cause of the hospitalisation, amputation, and disability burdens of people with diabetes [3, 4]. Diabetes-related foot ulceration is the most recognised DFD complication and people with diabetes have an estimated lifetime incidence of between 19% and 34% [5].

Prevention of DFD, particularly ulceration and amputation, have the potential to generate significant economic and social benefits for the international community [6]. In response, healthcare and research organisations worldwide have called for increasing investment in the prevention of DFD along with the development of international best practice guidelines for the prevention of DFD [7–9]. The International Working Group of the Diabetic Foot (IWGDF) guidelines are recognised as the DFD guidelines of highest quality and have been adopted and used in many countries of the world [10]. Implementation of IWGDF DFD guideline recommendations is associated with a decrease in the frequency of lower limb amputations [8]. However, despite the intention of guidelines to improve the quality of care and promote patient safety, it has been recognised that the publication of guidelines alone does not automatically lead to their application in practice [11]. In NZ, there are no official national DFD guidelines, except for a 2013 national risk stratification and referral pathway (New Zealand Society for the Study of Diabetes (NZSSD) risk stratification pathway) adapted from the Scottish Foot Action Group [12].

International guidelines also recognise that foot care provided by podiatrists is central to the prevention and management of DFD [13]. In NZ, approximately 21% of podiatrists report they work with people with diabetes, including 80% of those working primarily in private practice and approximately 8% in public diabetes-related foot services with the remainder in research or higher education settings [14]. Foot services for people with diabetes is frequently split between preventative care for people at increased risk of DFD and specialist services for management of people with DFD.

Given the importance of preventive care for the lower limb in people with diabetes, and the lack of NZ specific guidelines, the aim of this study was to determine the degree of alignment between the assessment and management strategies used by NZ podiatrists in the prevention of DFD and the 2019 IWGDF prevention guideline recommendations.

## Methods

### Study design and settings

This study was a cross-sectional observational designed study using an anonymous web-based survey. The study was approved by the Auckland University of Technology Ethics Committee (AUTEC 22/129) and the web-based survey was conducted between November 2 and December 14, 2022, using the Qualtrics XM, software package Provo, UT.

### Participants

All NZ registered podiatrists with a current annual practising certificate were eligible to participate. At the time of the survey closing there were 470 registered podiatrists in NZ (Registrar, Podiatrist’s Board of NZ, email on podiatrists with registration, 2023 Feb 18).

### Survey development/items collected

The design of the survey was adapted (with permission) based on a similar Australian-based survey [15], with the questions developed to align with the 2019 IWGDF prevention guidelines which were the most recent international guidelines at the time [8]. As it was considered that NZ podiatrists would be most familiar with the NZSSD risk stratification pathway, mapping was conducted to align the 2019 IWGDF prevention guideline recommendations and the NZSSD risk stratification pathway to improve face validity of the survey (Additional file 1). In this process, the prevention recommendations were used to develop survey questions that incorporated elements of both the NZSSD and IWGDF risk classification systems. A draft survey was piloted with four podiatrists, three from NZ and one from Australia. Pilot group respondents had diverse clinical backgrounds and experience in caring for people with diabetes-related foot disease in public and private practice. The draft survey was distributed through the Qualtrics platform via an anonymous survey link and all members of the pilot group completed the online survey and provided written feedback. Based on the feedback, questions and wording in the online survey were refined to create the final survey (Additional file 2). The final survey comprised 31-items covering the domains of participant characteristics (Q1-8), foot screening (Q9-11), identifying the at-risk foot (Q11-12), regularly inspecting and examining the at-risk foot (Q12), instructions on foot self-care (Q13), providing structured education about foot self-care (Q14), instructions about foot self-management (Q14), ensuring routine wearing of appropriate footwear (Q15), treatment of risk factors or pre-ulcerative signs on the foot (Q16-17), surgical interventions (Q25-27), foot-related exercises and weight-bearing activity (Q18-21), and integrated foot care (Q22).

### Procedure

An invitation to participate was distributed through professional podiatry networks in NZ including Facebook podiatry groups, the podiatry association, and email networks. Respondents followed an anonymous URL link and were directed to the participant information sheet, which detailed the purpose of the study, the duration of the survey, how the data would be stored, details of how anonymity was ensured, and the investigators’ contact details. Consent for participation was implicit with the submission of the survey. Anonymous responses were enabled in Qualtrics security settings to ensure respondents’ IP addresses, location data, and contact information were not recorded. Survey question back-tracking was enabled to allow respondents to review and change their answers, however, respondents were unable to make multiple survey submissions. A number of questions relating to clinical practice allowed for multiple selections. No survey question had a forced response requirement. A prize of one of five $100 coupons was also offered as an incentive to participate in the survey. Those participants responding to more than 50% of the survey questions/items were included in the final analysis.

### Data analysis

Descriptive statistics were used to display variable data using numbers and proportions for categorical data and median and interquartile range for ordinal data. The Mann Whitney U test was used to examine differences between subgroups. All analyses were undertaken in XLSTAT® software (version 2022.5.1) with a *p* value of < 0.05 considered significant. Survey data was reported in accordance with the Checklist for Reporting Results of Internet E-Surveys (Additional file 3) [16].

## Results

There were 77 total responses (16.3% of all NZ podiatrists with annual practising certificates). Fifty participants completed the survey in its entirety, with 4 participants completing between 50 and 95% of the survey. Of these, 2 participants did not submit any responses despite progressing through the survey and were excluded from the final analysis. Twenty-one participants (27%) completed less than 50% of the survey and were excluded from final data analysis. The total included responses analysed was 52 (67.5% of total participant responses).

 Table 1 displays the characteristics of the 52 included participants, including 73% from private practice, 50% based in Auckland and 49% identified as NZ European. Table 2 displays participant caseloads with respondents reporting treating a median of 21–30 patients with diabetes per week, including a median of 1–5-foot ulcers. Public podiatrists treated more people with foot ulcers per week (21-30) than private podiatrists (1-5) (*p* < 0.001). Table 3 shows the guidelines most commonly used to inform practice were the NZSSD risk stratification pathway (*n* = 32, 62%) and the IWGDF guidelines (*n* = 24, 46%).

**Table 1:** Participant characteristics

		Total	Private	Public	*P* value
**Work duration, n (%)**	0-2 years	10 (19)	10 (25)	0 (0)	**<0.001**
	3-5 years	7 (14)	5 (12)	2 (18)	0.62
	6-10 years	4 (8)	2 (5)	2 (18)	0.25
	11-15 years	9 (17)	7 (17)	2 (18)	0.81
	16-20 years	2 (4)	1 (2)	1 (9)	0.42
	21-25 years	10 (19)	7 (17)	3 (27)	0.49
	>26 years	10 (19)	9 (22)	1 (9)	0.56
**Ethnicity, n (%)**	NZ European/Pākehā	28 (49)	24 (52)	4 (37)	0.53
	Indian	7 (12)	7 (15)	0 (0)	0.07
	European	5 (9)	2 (4)	3 (27)	0.21
	Chinese	3 (5)	1 (2)	2 (18)	0.38
	Other Asian	4 (7)	4 (9)	0 (0)	0.46
	Other Ethnicity	3 (5)	3 (7)	0 (0)	0.80
	Southeast Asian	3 (5)	3 (7)	0 (0)	0.80
	Māori	2 (4)	2 (4)	0 (0)	0.21
	Latin American/Hispanic	1 (2)	0 (0)	1 (9)	0.69
	Middle eastern	1 (2)	0 (0)	1 (9)	0.69
**Geographical region, n (%)**	Auckland	26 (50)	21 (52)	5 (46)	0.91
	Canterbury	5 (9)	5 (12)	0 (0)	0.21
	Bay of Plenty	4 (8)	2 (5)	2 (18)	0.26
	Taranaki	4 (8)	4 (10)	0 (0)	0.23
	Waikato	4 (8)	3 (7)	1 (9)	0.78
	Wellington	5 (9)	3 (7)	2 (18)	0.36
	Manawatū-Whanganui	2 (4)	2 (5)	0 (0)	0.27
	Hawkes Bay	1 (2)	0 (0)	1 (9)	0.21
	Otago	1 (2)	1 (2)	0 (0)	0.30
**Work type, n (%)**	Private practice	38 (73)			
	High Risk Foot Service	11 (21)			
	Education	1 (2)			
	Research	1 (2)			
	Other	1 (2)

**Table 2:** Diabetes caseload

	Total	Private	Public	*P* value
**People with diabetes treated/week, median (IQR)**	11-20 (1-5 - 21-30)	11-20 (1-5 - 21-30)	31-40 (21-30 - >51)	**0.03**
**People with diabetic foot ulcers treated/week, median (IQR)**	1-5 (0 - 6-10)	1-5 (0 - 1-5)	21-30 (11-20 - 31-40)	**< 0.001**

**Table 3:** Guidelines utilised in practice

		Total n (%)	Private n (%)	Public n (%)	*P* value
Information sources used to guide assessment and management of people with diabetes^#^	NZSSD	32 (62)	24 (30)	8 (15)	0.06
	IWGDF	24 (46)	14 (18)	10 (19)	0.91
	Health Navigator NZ	9 (17)	5 (6)	4 (7)	0.81
	DFA	9 (17)	2 (3)	7 (13)	0.06
	NICE	9 (17)	3 (4)	6 (11)	0.16
	bpac	8 (15)	4 (5)	4 (7)	0.61
	SIGN	8 (15)	4 (5)	4 (7)	0.61
	Goodfellow Unit	8 (15)	5 (6)	3 (6)	0.92
	IDF	5 (10)	1 (1)	4 (7)	0.13
	ADA	5 (10)	2 (3)	3 (6)	0.47
	Other	5 (10)	4 (5)	1 (2)	0.49
	None of the above	11 (21)	11 (14)	0 (0)	**0.01**

*NZSSD *New Zealand Society for the Study of Diabetes, *IWGDF *International Working Group for the Diabetic Foot, *DFA *Diabetes Feet Australia, *NICE *National Institute for Health and Care Excellence, *bpac *Best Practice Advocacy Centre New Zealand, *SIGN *Scottish Intercollegiate Guidelines Network, *IDF *International Diabetes Federation, *ADA *American Diabetes Association

 Table 4 shows screening frequency and diagnostic tests utilised. The frequency of screening of the very low risk foot (IWGDF 0) was performed annually as recommended by the IWGDF guidelines by 44% of respondents, with the remainder indicating that they screen more frequently (29%) or only at initial consult (27%). The screening frequency of the low-risk foot (IWGDF 1) was performed every 6–12 months as recommended by the IWGDF guidelines by 18% of respondents, with the screening of the moderate-high risk foot (IWGDF 2–3) between 1 and 6 months by 78% of respondents. Of the screening diagnostic tests used, private podiatrists used Doppler examination without waveform analysis more often when assessing peripheral artery disease (*p* = 0.01) and public podiatrists used the Ipswich touch test more frequently when assessing peripheral neurological supply (*p* = 0.02).

**Table 4:** Participant characteristics

			Total	Private	Public	*P* value
**Screening frequency of the very low, moderate, and high-risk foot**^a^	**Screening of the very low risk (IWGDF 0) foot n (%)**	Initial consult only	14 (27)	12 (32)	1 (9)	0.14
		Every 1-3 Months	0 (0)	0 (0)	0 (0)	1.00
		Every 3-6 Months	4 (8)	2 (5)	2 (18)	0.56
		Every 6-12 Months	8 (15)	5 (13)	1 (9)	1.00
		Annually	23 (44)	18 (47)	6 (54)	0.94
		Never	3 (6)	1 (3)	1 (9)	0.95
	**Screening of the low risk foot (IWGDF 1) n (%)**	Initial consult only	2 (4)	2 (5)	0 (0)	0.25
		Every 1-3 Months	7 (14)	3 (8)	4 (36)	0.06
		Every 3-6 Months	21 (43)	17 (45)	4 (36)	0.85
		Every 6-12 Months	9 (18)	7 (18)	2 (18)	0.86
		Annually	10 (20)	9 (24)	1 (9)	0.43
		Never	0 (0)	0 (0)	0 (0)	1.00
	**Screening of the moderate-high risk foot (IWGDF 2-3) n (%)**	Initial consult only	0 (0)	0 (0)	0 (0)	1.00
		Every 1-3 Months	24 (49)	16 (42)	8 (73)	0.11
		Every 3-6 Months	14 (29)	13 (34)	1 (9)	0.10
		Every 6-12 Months	5 (10)	3 (8)	2 (18)	0.39
		Annually	6 (12)	6 (16)	0 (0)	0.09
		Never	0 (0)	0 (0)	0 (0)	1.00
**Diagnostic tests utilised in practice (peripheral vascular supply)**^b^		Manual palpation of pulses	52 (100)	37 (100)	11 (100)	0.96
		Capillary refill time/SVPFT	44 (85)	22 (59)	9 (82)	0.98
		Temperature gradient	35 (67)	32 (86)	9 (82)	0.56
		Doppler examination (with waveform)	27 (52)	16 (43)	10 (91)	0.14
		Doppler examination (without waveform)	20 (38)	18 (49)	1 (9)	**0.01**
		Toe systolic pressure (absolute toe pressure)	14 (27)	5 (14)	6 (55)	0.20
		Ankle brachial index	12 (23)	3 (8)	5 (45)	0.29
		Toe brachial index	9 (17)	7 (19)	5 (45)	0.14
		Ankle systolic pressure	5 (10)	1 (3)	3 (27)	0.10
		Other	5 (10)	1 (3)	3 (27)	0.10
**Diagnostic tests utilised in practice (peripheral neurological supply)**^b^		10g Monofilament	52 (100)	37 (100)	11 (100)	0.78
		Sharp/Blunt	31 (60)	21 (57)	7 (64)	0.72
		Hot/Cold	26 (50)	18 (49)	7 (64)	0.85
		128Hz Tuning fork	25 (48)	17 (46)	5 (45)	0.79
		Light Touch	25 (48)	16 (43)	6 (55)	0.86
		Joint Position Test	25 (48)	16 (43)	5 (45)	0.84
		Reflexes (Achilles/patella)	21 (40)	15 (41)	4 (36)	0.83
		Diabetic Neuropathy Symptom Score	14 (27)	8 (22)	4 36)	0.62
		Biosthesiometer	11 (21)	6 (16)	3 (27)	0.72
		Ipswich Touch Test	10 (19)	4 (11)	6 (55)	**0.02**
		Two Point Discrimination	5 (10)	4 (11)	0 (0)	0.70
		Other:	2 (4)	1 (3)	0 (0)	0.26

^a^Low risk foot (IWGDF 1) in gory in order to align the IWGDF risk stratification system with the NZSSD risk stratification system widely utilised within New Zealand. ^b^Participants were able to select multiple answers

 Table 5 displays the median (interquartile range) results for assessment and management of the low, moderate, and high-risk foot. Participants registered median scores of at least “often” (< 2) for all items in the frequency of assessment and management, inspection and examination of the high-risk foot, and frequency of education provision domains. High Risk Foot Service (HRFS) podiatrists reported more prescribing of custom-made footwear for the moderate to high-risk foot (*p* = 0.04) and more care as part of a multidisciplinary foot team more often that private podiatrists (*p* = 0.03). Participants indicated that they sometimes provide foot and mobility related exercises and often encourage daily walking, however when prompted to answer an open-ended question around what resources or guidance they used to help guide this provision, no responses were received.

**Table 5:** Assessment and management of a person with diabetes

			Total	Private	Public	*P* value
**Frequency that assessment guided by evidence-based guidelines**			2 (1-2)	2 (1-2)	1 (1-2)	0.30
**Frequency that management guided by evidence-based guidelines**			1 (1-2)	2 (1-2)	1 (1-2)	0.41
**Frequency of inspection & examination of the at-risk foot**	History of foot ulceration		1 (1-1)	1 (1-2)	1 (1-1)	0.27
	History of lower extremity amputation		1 (1-1)	1 (1-1)	1 (1-1)	0.68
	Diagnosis of end-stage renal disease		1 (1-2)	1 (1-3)	1 (1-2)	0.06
	Presence or progression of foot deformity		1 (1-1)	1 (1-2)	1 (1-1)	0.17
	Limited joint mobility		1 (1-2)	1 (1-2)	2 (1-2)	0.30
	Significant callus		1 (1-1)	1 (1-1)	1 (1-1)	0.84
	Pre-ulcerative signs		1 (1-1)	1 (1-1)	1 (1-1)	0.60
**Frequency of education provision relating to the prevention of DFD**	Foot care	Medium risk	1 (1-1)	1 (1-1)	1 (1-1)	0.84
		High risk	1 (1-1)	1 (1-1)	1 (1-1)	0.84
	Foot hygiene	Medium risk	1 (1-2)	1 (1-2)	1 (1-2)	0.30
		High risk	1 (1-2)	1 (1-2)	1 (1-1)	0.53
	Footwear	Medium risk	1 (1-2)	1 (1-2)	1 (1-2)	0.52
		High risk	1 (1-1)	1 (1-2)	1 (1-1)	0.60
	First aid	Medium risk	1 (1-3)	2 (1-2)	1 (1-3)	0.18
		High risk	1 (1-2)	2 (1-3)	1 (1-1)	0.21
**Frequency of provision of prescription of footwear and orthotic interventions to patients at moderate to high risk of DFD with a significant foot deformity**	Therapeutic footwear		2 (2-3)	3 (2-3)	2 (1-2)	0.23
	Custom-made footwear		3 (3-4)	4 (3-4)	2 (1-3)	**0.04**
	Custom-made orthoses		3 (2-3)	3 (2-4)	2 (1-2)	0.12
	Prefabricated insoles		3 (2-3)	3 (2-3)	2 (2-4)	0.12
	Toe orthoses		3 (2-3)	2 (2-3)	3 (2-4)	0.11
**Frequency of treatment of people at moderate to high risk of DFD**	Treat a pre-ulcerative sign or significant callus		2 (1-1)	2 (1-2)	1 (1-2)	0.27
	Treat ingrown toenails		2 (1-3)	2 (1-2)	1 (1-2)	0.41
	Treat fungal infections of the foot		2 (1-3)	2 (1-2)	3 (1-4)	0.48
**Frequency of provision of exercise and mobility for people with diabetes who were at Low to Moderate Risk of DFD**	Prescribe foot and mobility-related exercises		3 (3-4)	3 (2-4)	3 (3-4)	0.74
	Encourage daily walking		2 (1-2)	2 (1-2)	2 (2-3)	1.00
**Frequency of provision of integrated healthcare for people with diabetes**	Provide care as part of a multi-disciplinary team		3 (2-3)	3 (2-4)	2 (1-3)	**0.03**
	Provide telehealth services		5 (4-5)	5 (4-5)	5 (3-5)	0.46
	Provide care in remote locations		5 (3-5)	5 (3-5)	5 (4-5)	0.14
	Collaborate with Māori health providers		4 (3-5)	5 (3-5)	3 (2-4)	0.18
**Frequency of referral for surgical offloading interventions**			3 (1-3)	3 (1-5)	2 (1-3)	0.52
**Frequency of referral for a nerve decompression procedure**			5 (4-5)	5 (4-5)	5 (4-5)	0.49

Median Likert agreement value with interquartile range (IQR) for 5-point Likert scale responses; response options Always (1), Often (2), Sometimes (3), Seldom (4), Never (5); DFD, Diabetes-related foot disease

 Table 6 displays the education modalities employed, nearly all participants reported using verbal education to discuss the nature of diabetes (98%), preventative strategies (100%) and management (94%). The provision of structured education was only used by 5% of practitioners. There were no significant differences between HRFS and private podiatrists in the provision of education.

**Table 6:** Education provision

		1:1 verbal education n (%)				Resources and Handouts n (%)				Links to external support networks n (%)				Structured Education Programme n (%)			
**Frequency of education provision and modalities employed to achieve this**^a^		**Total**	**Private**	**Public**	***P*** **value**	**Total**	**Private**	**Public**	***P*** **value**	**Total**	**Private**	**Public**	***P*** **value**	**Total**	**Private**	**Public**	***P*** **value**
	The nature and effect of diabetes on the lower limb, n (%)	51 (98)	40 (98)	11 (100)	0.99	21 (40)	16 (38)	5 (45)	0.67	10 (19)	7 (17)	3 (27)	0.49	4 (8)	4 (10)	0 (0)	0.19
	Preventative foot care behaviours, including hygiene and foot inspection	51 (100)	41 (100)	10 (91)	0.70	19 (37)	15 (37)	4 (36)	0.89	9 (18)	7 (17)	2 (18)	0.79	2 (4)	2 (5)	0 (0)	0.26
	Management of special problems (particular to the patient)	47 (92)	37 (90)	10 (91)	0.90	22 (44)	17 (41)	5 (12)	0.78	6 (12)	5 (12)	1 (9)	0.97	4 (8)	3 (7)	1 (9)	0.75

^a^Participants were able to select multiple answers for this question

Table 7 presents referral options and barriers reported to accessing referral options. For foot ulcer referrals, 98% reported having access to general practitioner (GP) services, 88% multidisciplinary diabetes foot services, 63% vascular surgery and 58% orthopaedic options available for referrals, with no significant difference between public and private practitioners. In terms of barriers to provision of preventative care, 73% identified resource barriers such as service availability, staffing and wait times for clinicians accessing referral options for people with active foot ulcers, 68% (*n* = 30) patient factor barriers, and 43% (*n* = 19) communication barriers.

**Table 7:** Referral options available and barriers to provision of care for an active ulceration or active risk foot

		Total n (%)	Private n (%)	Public n (%)	*P* value
**Referral/clinical support options available**^a^	Multi-disciplinary diabetic foot service	42 (88)	33 (87)	9 (90)	0.77
	Vascular Surgery Referral Pathway	30 (63)	22 (58)	8 (80)	0.28
	Orthopaedic Surgery Referral Pathway	28 (58)	21 (55)	7 (70)	0.47
	General Practitioner	47 (98)	38 (100)	9 (90)	0.70
**Barriers/constraints experienced when caring for a foot ulceration which is not responding to appropriate therapy**^a^	Communication barriers (including referrals, interdisciplinary connections, access to medical records)	19 (43)	15 (47)	4 (40)	0.89
	Resource barriers (including service availability, staffing and wait times)	32 (73)	27 (80)	5 (50)	0.40
	Practitioner knowledge barriers (including competence and confidence)	11 (25)	8 (20)	3 (30)	0.60
	Patient factor barriers (understanding, socioeconomic factors and geographical location)	30 (68)	20 (67)	7 (70)	0.45
	Other	15 (34)	12 (37)	3 (30)	0.94

^a^Participants were able to select multiple answers

## Discussion

This study was the first to survey NZ podiatrists on their practices related to the prevention of DFD. The results indicate that the screening of the very low to moderate risk foot was undertaken more frequently than guideline recommendations, but that the screening of the high-risk foot was in alignment with international guidelines. Podiatrists indicated that they were generally applying recommendations for instructions on foot self-care, foot self-management, and treatment of risk factors or pre-ulcerative signs on the foot. Partial application of recommendations was found for the routine wearing of appropriate footwear, surgical interventions, foot-related exercise and weight-bearing activities, and integrated care. Only one recommendation on the provision of structured education was identified as not being applied in practice.

Less than half of respondents reported that they would screen the very low to moderate risk foot as per the guideline recommendation, indicating a high level of inconsistency between podiatrists in terms of screening frequency. This finding indicates that there may be an over screening of people with low or very low risk of DFD. Although there is evidence that screening prevents the development of DFD in the high-risk population, there is limited evidence that population screening reduces risk of DFD for all people with diabetes [17–19]. This reinforces the need for the development of national guidelines for the prevention of DFD.

In the diagnostic tests employed by podiatrists in order to identify the at-risk foot, our findings showed that all respondents were consistent with the recommendations of both the 2019 IWGDF prevention guidelines and the NZSSD risk stratification system [20]. Manual pulse palpation continues to be the most frequently employed vascular assessment employed by podiatrists, despite concerns around its accuracy, interpretation and prognostic utility in detecting the presence of peripheral arterial disease [21, 22]. This finding is consistent with a similar survey by Tehan [23], which identified that podiatrists in Australia and NZ continue to rely on subjective vascular assessment testing methods such as pedal pulse palpation, over objective measurements such as the ankle brachial index (ABI) and toe brachial index (TBI). In the application of tests relating to the detection of peripheral neuropathy, all respondents indicated the utilisation of the 10 g monofilament in clinical practice, which has been found to provide the most consistent results in the prediction of foot ulceration [22].

In relation to education provision, podiatrists appear to be mostly providing this through 1:1 verbal education, with the provision of structured education the least used form of all patient education modalities. The quality of evidence that structured education alone is effective in achieving clinically relevant reductions in foot ulcer risk is low, with a lack of association between structured education and clinically meaningful reductions in foot ulcer risk reported [24]. However, the IWGDF prevention guidelines recommends structured education as preferable to other educational modalities as part of a larger movement away from didactic models of care in which the patient is a passive recipient of standardised information, and towards the integration of psychosocial model and patient centred programs [25–27]. The survey findings are consistent with previous research which has shown that implementing diabetes self-management education into routine clinical care can be challenging, as much of diabetes management centres around changing the behaviours of the individual with often multidimensional risk factors [28–30].

Education and encouragement of exercise and daily walking was found to be often recommended in clinical practice. Despite NZ podiatrists advocating for the importance of physical exercise and mobility, the results indicate that the education provided to patients around exercise is still largely based on the clinician’s individual experience. Exercise has been identified as potentially playing an important role as an intervention in the non-pharmacological treatment of DFD, including on the progression of diabetes-related peripheral neuropathy [31]. However, despite an increased number of studies investigating the provision of foot and mobility-related exercise as an intervention to prevent foot ulcers there continues to be a small research evidence base in this area [24, 32]. The findings of our survey may indicate that clinicians need further support in the application of research in the provision of foot-related exercises and weight-bearing activity in the prevention of DFD.

The results surrounding multidisciplinary teams indicate there is widespread establishment in NZ, but the delivery of preventive care more broadly through other integrated modalities of care such as Māori healthcare providers and telemedicine remains limited. For people with diabetes, integrated care has the potential to improve outcomes, disability, morbidity, and mortality, with the utilisation of integrated health being associated with a reduction in first presentations of diabetes-related foot ulcerations [33, 34].

Partial application of footwear recommendations was identified, with more podiatrists utilising off-the-shelf therapeutic over custom-made footwear. Podiatrists in NZ indicated the preferential use of prefabricated insoles over custom insoles. This is consistent with the evidence on orthotic interventions, with previous research identifying a positive association between the use of therapeutic footwear and foot orthotics in foot ulcer prevention [35].

These survey findings should be interpreted in respect to limitations. Firstly, the sampling technique may have resulted in sampling bias. As the study undertaken was voluntary and entitled ‘diabetic foot care research’ and promoted through public health networks as well as through social media, it may be that most respondents were podiatrists who had experience in, or an interest with, the care of people with diabetes. The respondents to our survey were found to be broadly representative of the private podiatry workforce (73% versus 80% in overall employment), with a higher proportion of public podiatrists responding (21% versus 8% in the overall employment) [14]. Secondly, using a non-validated survey tool decreases the reliability and external validity of our results. This limitation was minimised by the basing the concept and questions on a similar survey undertaken by Quinton et al. in Australia [15] and referencing several questions and wording from the IWGDF prevention guideline [25]. It was further minimised by the undertaking of piloting with a small sample of experienced podiatric clinicians. Thirdly, the study had a low response rate (16% of NZ podiatrists with annual practising certificates). However, this response rate is approximately double that than the 8% reported by Quinton et al. [15] in their similar study on diabetes-related foot assessment practices of podiatrists in Australia and slightly higher than Yuncken et al. [29] who had a 10% response rate in a survey of podiatrists on the provision of education to people with diabetes. Additionally, previous research has identified that only a small percentage of the podiatry profession in NZ work primarily with people with diabetes on a daily basis (22%) [14] which may have contributed to the low response rate. Finally, approximately 50% of the respondents to this survey were from Auckland compared to regional areas of NZ. This may be a consideration when interpreting findings particularly to those relating to the access and availability of referral avenues and healthcare resources.

## Conclusion

This study presents the first known data collected on the assessment and management used by NZ podiatrists to prevent diabetes-related foot disease. NZ podiatrists generally follow international guideline recommendations with respect to the examination of the at-risk foot, instructions on foot self-care, appropriate footwear and treatment of risk factors and pre-ulcerative signs, however there may be some over screening of the low-risk foot occurring in clinical practice. Increasing access to integrated healthcare, implementing structured educational programmes, and supporting clinicians in the provision of exercise and weight-bearing activities in people with diabetes appear to be areas of practice that need future development in NZ.

### Supplementary Information

**Additional file 1.**

**Additional file 2.**

**Additional file 3.**

## Data Availability

The responses that individually support the results are not available publicly due to confidentiality.

## Abbreviations

ABI: Ankle brachial index
AUTEC: Auckland University of Technology
DFD: Diabetes-related foot disease
ESRD: End stage renal disease
GP: General practitioner
HRFS: High-Risk Foot Service
IWGDF: International Working Group on the Diabetic Foot
NZ: Aotearoa New Zealand
NZSSD: New Zealand Society for the Study of Diabetes
PAD: Peripheral arterial disease
TBPI: Toe brachial pressure index
