# Supplementary material for: How does the clinical practice of Aotearoa New Zealand podiatrists align with international guidelines for the prevention of diabetes-related foot disease? A cross-sectional survey
Source: J Foot Ankle Res. 2023 Aug 22;16:53. doi: 10.1186/s13047-023-00651-x (PMC10464278; doi:10.1186/s13047-023-00651-x)
Supplement: Supplementary file 1 — Additional file 1. [file 13047_2023_651_MOESM1_ESM.docx]

**How does the clinical practice of Aotearoa New Zealand podiatrists align with international guidelines for the prevention of diabetic foot disease? a cross-sectional survey**

**Supplementary file 1**

New Zealand utilises the New Zealand Society for the Study of Diabetes (NZSSD) risk stratification system which is adapted with permission from the Scottish Intercollegiate Guidelines Network (SIGN) 2016 Diabetic Foot Risk Stratification and Triage System. An alignment table between IWGDF 2019 recommendations and NZSSD risk stratification was developed, and the recommendations were written to ensure that there was reasonable consistency between the two risk stratification systems. Both systems had a similar criterion for IWGDF Very Low Risk (IWGDF 0) and NZSSD Low Risk, however for the NZSSD Moderate Risk, the alignment of this fell between IWGDF Low risk and Moderate Risk (IWGDF 1-2) and for NZSSD High Risk, alignment of this fell between IWGDF Moderate Risk and IWGDF High Risk (IWGDF 2-3) *(see figure 1).*


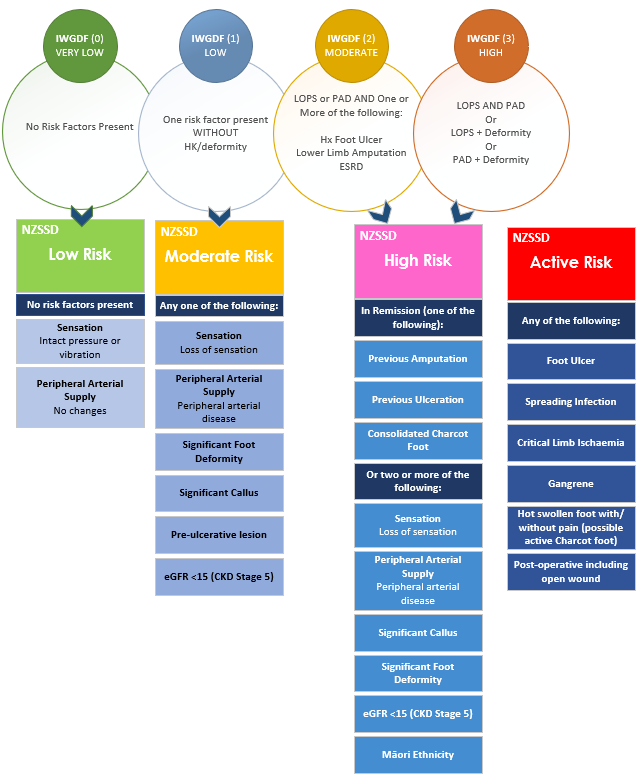


Figure 1 Alignment Diagram
