# Supplementary material for: How does the clinical practice of Aotearoa New Zealand podiatrists align with international guidelines for the prevention of diabetes-related foot disease? A cross-sectional survey
Source: J Foot Ankle Res. 2023 Aug 22;16:53. doi: 10.1186/s13047-023-00651-x (PMC10464278; doi:10.1186/s13047-023-00651-x)
Supplement: Supplementary file 2 — Additional file 2. [file 13047_2023_651_MOESM2_ESM.docx]

**How does the clinical practice of Aotearoa New Zealand podiatrists align with international guidelines for the prevention of diabetic foot disease? a cross-sectional survey**

### Supplementary file two

Questions mapped to IWGDF recommendations.

**Identifying the at-risk foot**

Recommendation #1

*Examine a person with diabetes at very low risk of foot ulceration (IWGDF risk 0) annually for signs or symptoms of loss of protective sensation and peripheral artery disease, to determine if they are at increased risk for foot ulceration. (GRADE recommendation: Strong; Quality of evidence: High)*


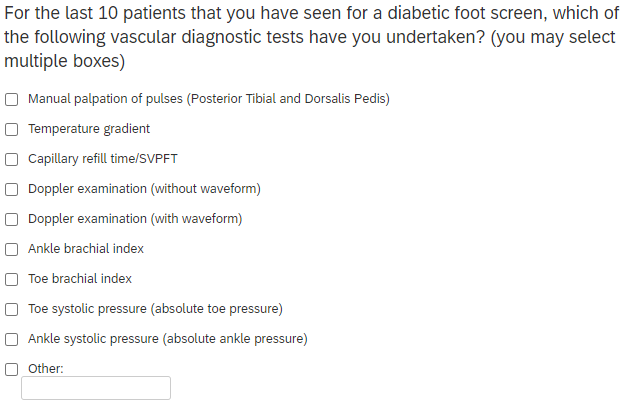


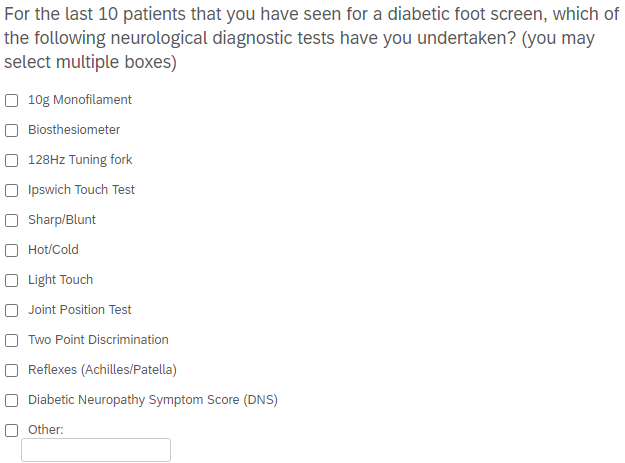


**Regularly inspecting and examining the at-risk foot**

Recommendation #2

*Screen a person with diabetes at risk of foot ulceration (IWGDF risk 1-3) for:*

- *a history of foot ulceration or lower-extremity amputation;*
- *diagnosis of end-stage renal disease;*
- *presence or progression of foot deformity;*
- *limited joint mobility;*
- *abundant callus; and*
- *any pre-ulcerative sign on the foot.*

*Repeat this screening once every 6-12 months for those classified as IWGDF risk 1, once every 3-6 months for IWGDF risk 2, and once every 1-3 months for IWGDF risk 3. (Strong; High)*


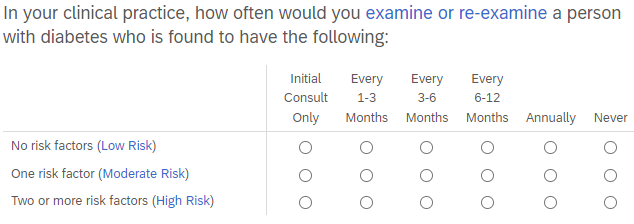


*
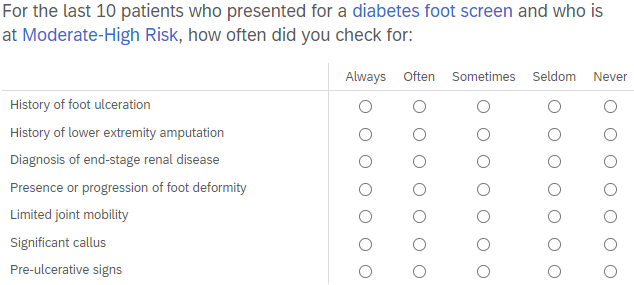
*

**Instructions on foot self-care**

Recommendation #3

*Instruct a person with diabetes who is at risk of foot ulceration (IWGDF risk 1-3) to protect their feet by not walking barefoot, in socks without shoes, or in thin-soled slippers, whether indoors or outdoors. (Strong; Low)*

*
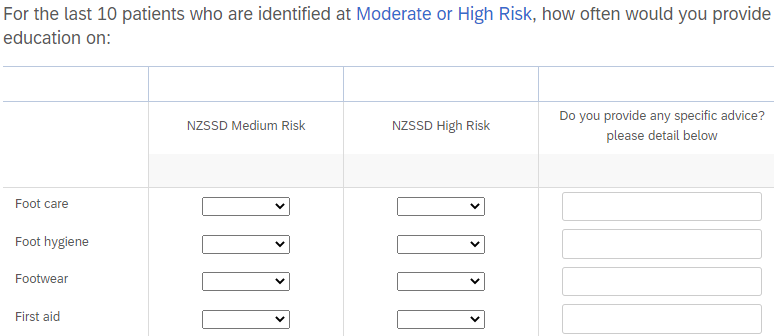
*

Recommendation #4

*Instruct, and after that encourage and remind, a person with diabetes who is at risk of foot ulceration (IWGDF risk 1-3) to: inspect daily the entire surface of both feet and the inside of the shoes that will be worn; wash the feet daily (with careful drying, particularly between the toes); use emollients to lubricate dry skin; cut toenails straight across; and, avoid using chemical agents or plasters or any other technique to remove callus or corns. (Strong; Low)*

**Providing structured education about foot self-care**

# Recommendation #5

*Provide structured education to a person with diabetes who is at risk of foot ulceration (IWGDF risk 1-3) about appropriate foot self-care for preventing a foot ulcer. (Strong; Low)*

*
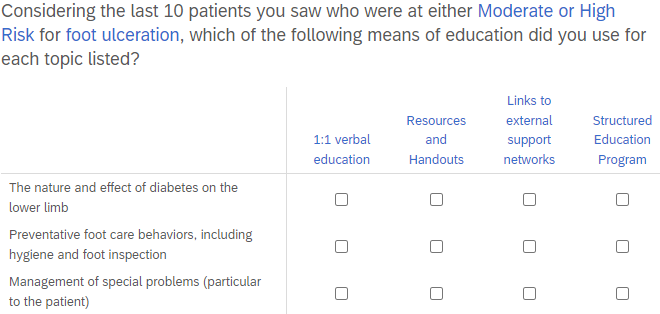
*

**Instructions about foot self-management**

Recommendation #6

*Consider instructing a person with diabetes who is at moderate or high risk of foot ulceration (IWGDF risk 2-3) to self-monitor foot skin temperatures once per day to identify any early signs of foot inflammation and help prevent a first or recurrent plantar foot ulcer. If the temperature difference is above-threshold between similar regions in the two feet on two consecutive days, instruct the patient to reduce ambulatory activity and consult an adequately trained health care professional for further diagnosis and treatment. (Weak, Moderate)*

This recommendation was not included in the survey as the technology has not become widespread for its use.

**Ensuring routine wearing of appropriate footwear**

Recommendation #7

*Instruct a person with diabetes who is at moderate risk for foot ulceration (IWGDF risk 2) or who has healed from a non-plantar foot ulcer (IWGDF risk 3) to wear therapeutic footwear that accommodates the shape of the feet and that fits properly, to reduce plantar pressure and help prevent a foot ulcer. When a foot deformity or a pre-ulcerative sign is present, consider prescribing custom-made footwear, custom-made insoles, or toe orthoses. (Strong; Low)*

*
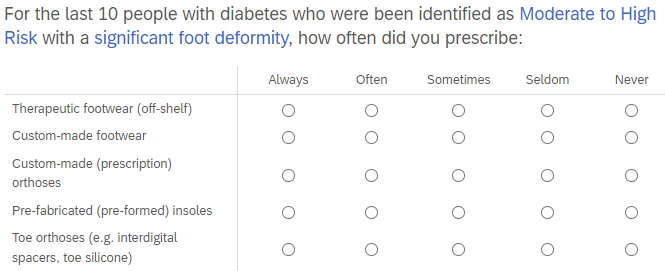
*

Recommendation #8

*Consider prescribing orthotic interventions, such as toe silicone or (semi-)rigid orthotic devices, to help reduce abundant callus in a person with diabetes who is at risk for foot ulceration (IWGDF risk 1-3). (Weak; Low)*

Recommendation #9

*In a person with diabetes who has a healed plantar foot ulcer (IWGDF risk 3), prescribe therapeutic footwear that has a demonstrated plantar pressure relieving effect during walking, to help prevent a recurrent plantar foot ulcer; furthermore, encourage the patient to consistently wear this footwear. (Strong; Moderate)*

*
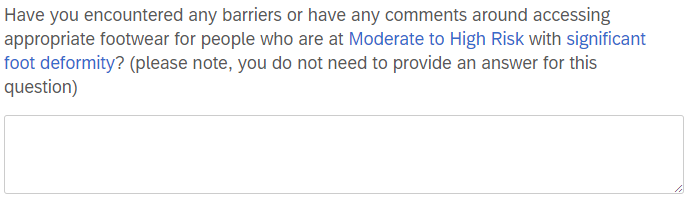
*

**Treatment of risk factors or pre-ulcerative signs on the foot**

Recommendation #10

*Treat any pre-ulcerative sign or abundant callus on the foot, ingrown toenail, and fungal infection on the foot, to help prevent a foot ulcer in a person with diabetes who is at risk of foot ulceration (IWGDF risk 1-3). (Strong; Low)*

*
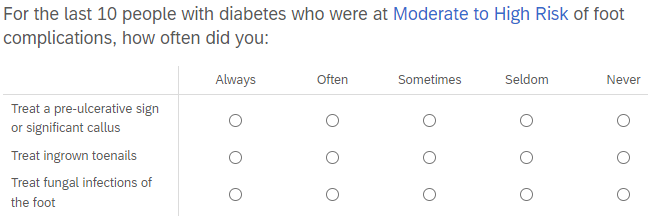
*

**Surgical Interventions**

Recommendation #11

*In a person with diabetes and abundant callus or an ulcer on the apex or distal part of a non-rigid hammertoe that has failed to heal with non-surgical treatment, consider digital flexor tendon tenotomy for preventing a first foot ulcer or recurrent foot ulcer once the active ulcer has healed (Weak; Low).*

Recommendation #12

*In a person with diabetes and a plantar forefoot ulcer that has failed to heal with non-surgical treatment, consider Achilles tendon lengthening, single or pan metatarsal head resection, metatarsophalangeal joint arthroplasty or osteotomy, to help prevent a recurrent plantar forefoot ulcer once the active ulcer has healed. (Weak; Low)*

*
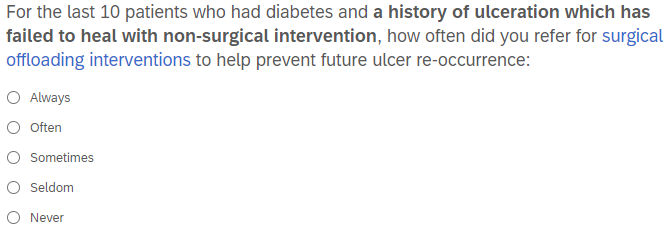
*

*
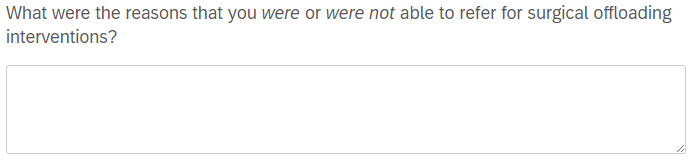
*

Recommendation #13

*We suggest not to use a nerve decompression procedure, in preference to accepted standards of good quality care, to help prevent a foot ulcer in a person with diabetes who is at moderate or high risk of foot ulceration (IWGDF risk 2-3) and who is experiencing neuropathic pain. (Weak; Low)*

*
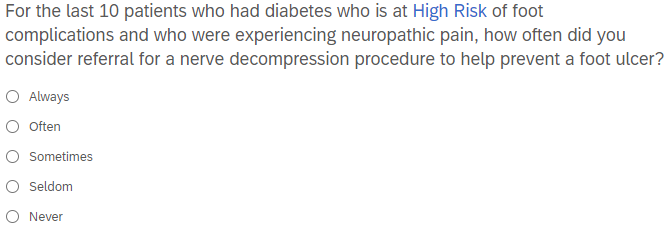
*

**Foot-related exercises and weight-bearing activity**

Recommendation #14

*Consider advising a person with diabetes who is at low or moderate risk for foot ulceration (IWGDF risk 1 or 2) to perform foot and mobility-related exercises with the aim of reducing risk factors of ulceration, that is, decreasing peak pressure and increasing foot and ankle range of motion, and with the aim of improving neuropathy symptoms. (Weak; Moderate)*

*
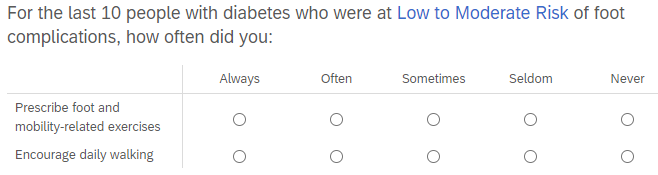
*

*
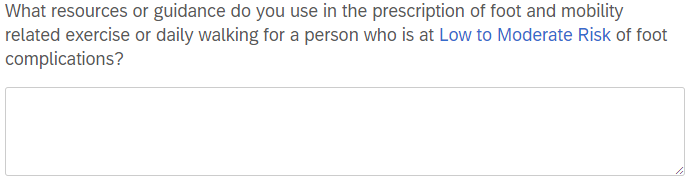
*

*
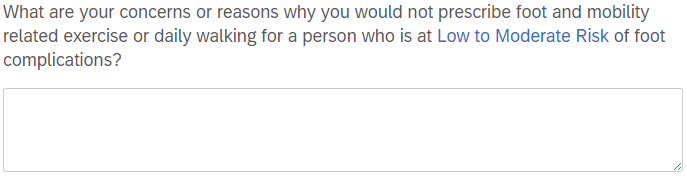
*

Recommendation #15

*Consider communicating to a person with diabetes who is at low or moderate risk for foot ulceration (IWGDF risk 1 or 2) that a moderate increase in the level of walking-related weight-bearing activity (ie, an extra 1.000 steps/day) is likely to be safe. Advise this person to wear appropriate footwear when undertaking weight-bearing activities, and to frequently monitor the skin for pre-ulcerative signs or breakdown. (Weak; Low)*

*
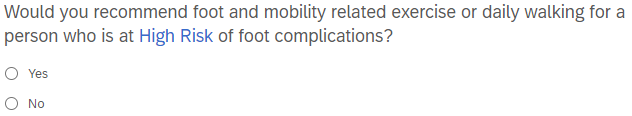
*

**Integrated foot care**

Recommendation #16

*Provide integrated foot care for a person with diabetes who is at high risk of foot ulceration (IWGDF risk 3) to help prevent a recurrent foot ulcer. This integrated foot care includes professional foot care, adequate footwear and structured education about self-care. Repeat this foot care or re-evaluate the need for it once every one to three months, as necessary. (Strong; Low)*

*
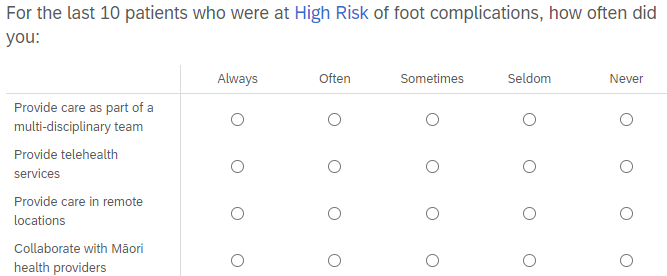
*
